# Mining and Analysis of SNP in Response to Salinity Stress in Upland Cotton (*Gossypium hirsutum* L.)

**DOI:** 10.1371/journal.pone.0158142

**Published:** 2016-06-29

**Authors:** Xiaoge Wang, Xuke Lu, Junjuan Wang, Delong Wang, Zujun Yin, Weili Fan, Shuai Wang, Wuwei Ye

**Affiliations:** State Key Laboratory of Cotton Biology, Institute of Cotton Research of Chinese Academy of Agricultural Sciences, Key Laboratory for Cotton Genetic Improvement, Anyang 455000, Henan, China; NBPGR, INDIA

## Abstract

Salinity stress is a major abiotic factor that affects crop output, and as a pioneer crop in saline and alkaline land, salt tolerance study of cotton is particularly important. In our experiment, four salt-tolerance varieties with different salt tolerance indexes including CRI35 (65.04%), Kanghuanwei164 (56.19%), Zhong9807 (55.20%) and CRI44 (50.50%), as well as four salt-sensitive cotton varieties including Hengmian3 (48.21%), GK50 (40.20%), Xinyan96-48 (34.90%), ZhongS9612 (24.80%) were used as the materials. These materials were divided into salt-tolerant group (ST) and salt-sensitive group (SS). Illumina Cotton SNP 70K Chip was used to detect SNP in different cotton varieties. SNPv (SNP variation of the same seedling pre- and after- salt stress) in different varieties were screened; polymorphic SNP and SNPr (SNP related to salt tolerance) were obtained. Annotation and analysis of these SNPs showed that (1) the induction efficiency of salinity stress on SNPv of cotton materials with different salt tolerance index was different, in which the induction efficiency on salt-sensitive materials was significantly higher than that on salt-tolerant materials. The induction of salt stress on SNPv was obviously biased. (2) SNPv induced by salt stress may be related to the methylation changes under salt stress. (3) SNPr may influence salt tolerance of plants by affecting the expression of salt-tolerance related genes.

## Introduction

Salinity stress is a major abiotic factor that affects crop output [1], and exploring the mechanism of salt tolerance of plants is of great importance for improving their salt tolerance and creating salt tolerant germplasms. As a pioneer crop in saline and alkaline land, salt tolerance study of cotton is particularly important.

SNP (Single nucleotide polymorphism) is genetic polymorphism caused by variation of single bases in different alleles at the same locus in the genome, and commonly SNP refers to conversion or transversion of single bases. Compared with other types of mutations, SNPs stand out by their high absolute numbers, biallelic nature, relatively low mutation rates and amenably automation, which allows high-throughput analyses of large numbers of samples [2]. With fully sequenced genomes are available, SNPs have been found in many species and microarrays with SNP markers (SNP-chip) have been developed for many plants [3–5]. These will contribute to the application of SNP markers in plants. Based on the location of occurrence, SNP could be divided into non-coding region SNP and coding region SNP (cSNP). A small portion of non-coding region SNP is located in gene control region, which is known as regulatory SNP (rSNP). According to their different effects on genetic traits, cSNP can be divided into two types: one is synonymous cSNP, which will not change the amino acid sequence of the protein being translated; another is non-synonymous cSNP, which refers to that changes in nucleotide sequence will cause the changes of downstream protein sequence. And SNP affecting the gene functions is called functional SNP (fSNP). In organisms, expression of the genes is a complex process. According to the central dogma, genetic information within the gene is transcribed to mRNA, translated and then transmitted to the polypeptide chain; after folding and assembly, polypeptide chain forms protein and play its functions. In this process, mutation of fSNP at certain key locus may cause changes in single chain folding [6], or lead to changes in the downstream amino acid sequence and nature, or make some restriction loci disappear or reappear, or change the gene structure and regulatory loci, and ultimately affect the phenotype. Researches also showed that certain link was found between SNP and methylation and epigenetic inheritance; documents also showed that 5mC at methylated CpG loci was prone to spontaneously deaminize and turned into thymine, and ultimately generated C/T or G/A mutation point after DNA replication [7]. Researches have shown that SNP can contribute to a phenotype directly or be associated with a phenotype [8].

Currently, a large number of fSNP associated with phenotypic traits have been found in animals and humans, and they have been comprehensively studied, while there are few studies on such fSNP in plants. A SNP was found on *CmOr* gene of orange flesh watermelon and green/white flesh watermelon and it caused an evolutionarily conserved arginine in CmOr protein to mutate into histidine, and studies found that the mutation would cause the accumulation of β-carotene, thus affecting the flesh colors of orange flesh watermelon and non-orange flesh watermelon [9]. Promoter region of *TaGW2-6A* gene of 207 Indian wheat varieties was sequenced and a new SNP site SNP -494 was discovered, which is related to grain weight and other agronomic traits such as ear length, protein content and so on. And this SNP is located in "CGCG" domain, which plays a key role in Ca^2+^/calmodulin mediated gene regulation [10]. A rare SNP was found on *qph1* gene in maize and it may affect plant height and was considered to possibly enhance the potential maize yield [11]. Plant nutrition-related SNP was also found. A nonsynonymous SNP was found on a 1.5kb DNA fragment containing *NRT1*.*1B* gene in rice, and numerous studies showed that this SNP could explain the differences of Indian rice and japonica rice in uptake activity of nitrate and the use efficiency of nitrogen [12]. Discovery of these fSNP showed that inheritance and variation of certain phenotypes takes SNP as a unit, thus to study these phenotypes through SNP is inevitable.

As for stress resistance, a series of stress-tolerance related SNP have been screened from stress-resistance related genes. Manish Roorkiwal et al. chose 10 abiotic stress related genes in chickpea as research subjects, and screened 79 SNPs and 41 indels from 9 genes [13]. Bharti Garg et al. screened a synonymous SNP that was related to dehydration stress at 458bp of *TaMYB2* gene in wheat [14]. These SNP finding in abiotic stress suggested that SNP played an important role in some complex quantitative traits, providing new methods and ideas for the study of stress resistance of plants. Plant salt tolerance was a complex quantitative trait, but there were few studies on salt tolerance-related SNP, and fSNP that impact salt tolerance was rarely reported. In our study, Illumina Cotton SNP 70K Chip (chip) was used to analyze SNPv in different varieties with different salt tolerance index pre- and after- salt stress and different SNP between four typical salt-tolerant materials and four typical salt-sensitive materials was screened so as to obtain potential SNP related to salt tolerance.

## Materials and Methods

### Preparation of materials and samples

Four typical salt-tolerant upland cotton (*Gossypium hirsutum* L.) varieties: CRI35, Kanghuangwei164, Zhong9807, CRI44 and four salt-sensitive varieties: Hengmian3 GK50, Xinyan96-48, ZhongS9612 from Institute of Cotton Research of CAAS were employed in this study. These varieties have been detected using 0.4% salinity stress method [15]. Based on the identification for years, the salt tolerance indexes of eight varieties in the study were shown in S1 Table. Seedlings were cultured in sand in the greenroom in Institute of Cotton Research of Chinese Academy of Agricultural Sciences. In three-leaf stage, seedlings were treated with 0.4% (1000g sand treated with 4g NaCl) of NaCl. Leaves were harvested from each plant of pre-salt stress and 48h after salt stress, frozen in liquid nitrogen and preserved at -80°C.

Plant DNA extraction kit (Bioteke, China) was used to extract DNA [16], according to the instruction of manufacturer. 1% agarose gel electrophoresis and nucleic acid analyzer (Nanodrop_2000) were used to detect quality and concentration of DNA. In detection of gel electrophoresis the gel hole was clean and free of residue, without smearing or severe degradation. DNA concentration was above 100ng/μl, which is in line with the chip testing requirements.

### SNP detection and genotyping

Cotton SNP 70K Chip (Illumina, USA) was used to detect SNP [17]. Samples of leaf tissues were collected from four salt-tolerant varieties and four salt-sensitive varieties pre- and after- salt treatments. Three biological replicates of every treatment were collected separately and 48 SNP chips were used in the study, finally. With the instruction of manufacturer, fragments of genome DNA were hybridized with the prepared SNP Chip. During hybridization, fragmented DNA complementarily paired with 50 bases which were connected to bead on the chip. With the captured DNA as the template, extension reaction of single base was carried out on the chip; detectable fluorophores were added to the chip so as to distinguish different SNP genotypes of the samples. GenomeStudio software was used to obtain SNP genotyping data of each sample.

### Screening and analysis of SNPv and SNPr

Screening of SNPv: eight materials were divided into salt tolerant group (ST) and salt sensitive group (SS). SNPv was obtained through comparison of SNP genotypes of single plant pre- and after- salt stress, and the same SNPv in ST group and SS group was taken as the ST-SNPv and SS-SNPv.

Screening of SNPr: Remove loci with different SNP types or more than two failing types in three biological replicates of each material, and the genotypes that appeared more than twice within three biological replicates were taken as the SNP genotype of the material. The SNP loci that have different SNP genotypes in different materials was the polymorphic SNP loci, upon which the SNP loci with the genotypes that were same within the group but different between the SS group and ST group (genotyping failure was allowed once in each group) were chosen as the potential SNPr.

Locations of SNP in genome were determined by alignment of probe of chip and the reference genome with bowtie (http://www.plob.org/2011/12/13/932.html). Qhysical map of SNP was constructed by Mapchart software, AgriGO (http://bioinfo.cau.edu.cn/agriGO/) was used for GO annotation of genes.

## Results and Analysis

### Statistics and analysis of SNPv pre- and after- salt stress

#### Statistics of SNPv in varieties with different salt tolerance index

**Fig 1:**
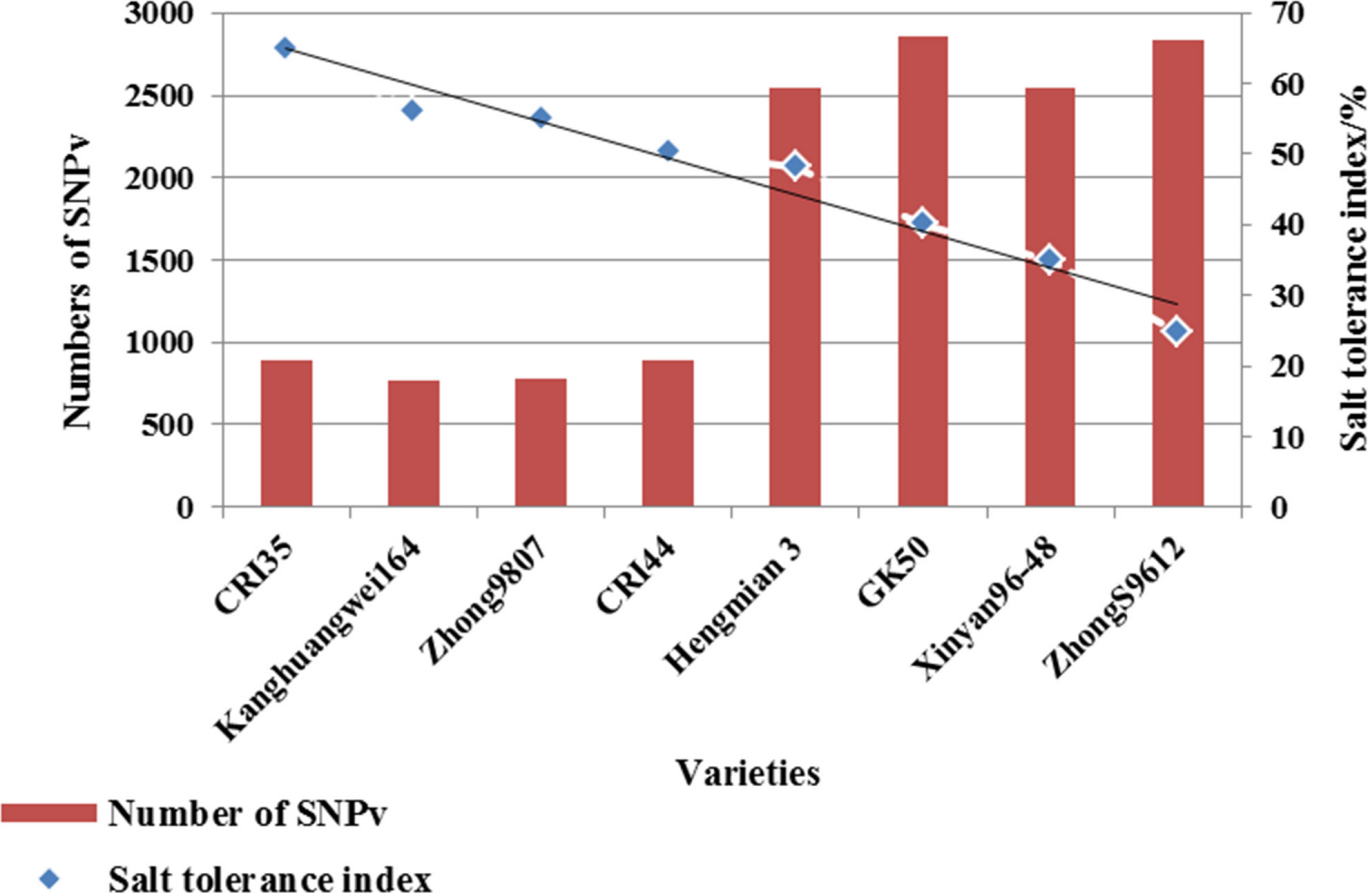
Salt tolerance index and numbers of SNPv in different varieties.

SNPv that appeared in three replicates were screened. The results showed that the number of SNPv in salt-tolerant materials was generally lower than that of salt-sensitive materials. The larger the salt tolerance index, the stronger the salt tolerance, the less SNPv (Fig 1). Further analysis showed that 429 SNPv appeared in three replicates of every variety in ST group, and among of them 414 appeared only in ST group; 1,365 SNPv appeared in three replicates of every variety in SS group, and among of them 1,350 appeared only in SS group (Table 1). In this experiment, three biological replicates significantly reduced SNP detection and genotyping errors, and results of SNPv loci appearing in three replicates are reliable.

**Table 1:** Statistics of SNPv numbers of different types.

SNPv types	SNPv in SS	Percents in SS/%	SNPv in ST	Percents in ST/%
A/C	137	9.45	46	11.11
G/A	555	38.28	179	43.24
C/T	514	35.45	151	36.47
T/G	144	9.93	38	9.18
Specific SNPv	1350		414	
SNPv	1365		429	

#### Distribution of SNPv sites on chromosomes in different varieties

**Fig 2:**
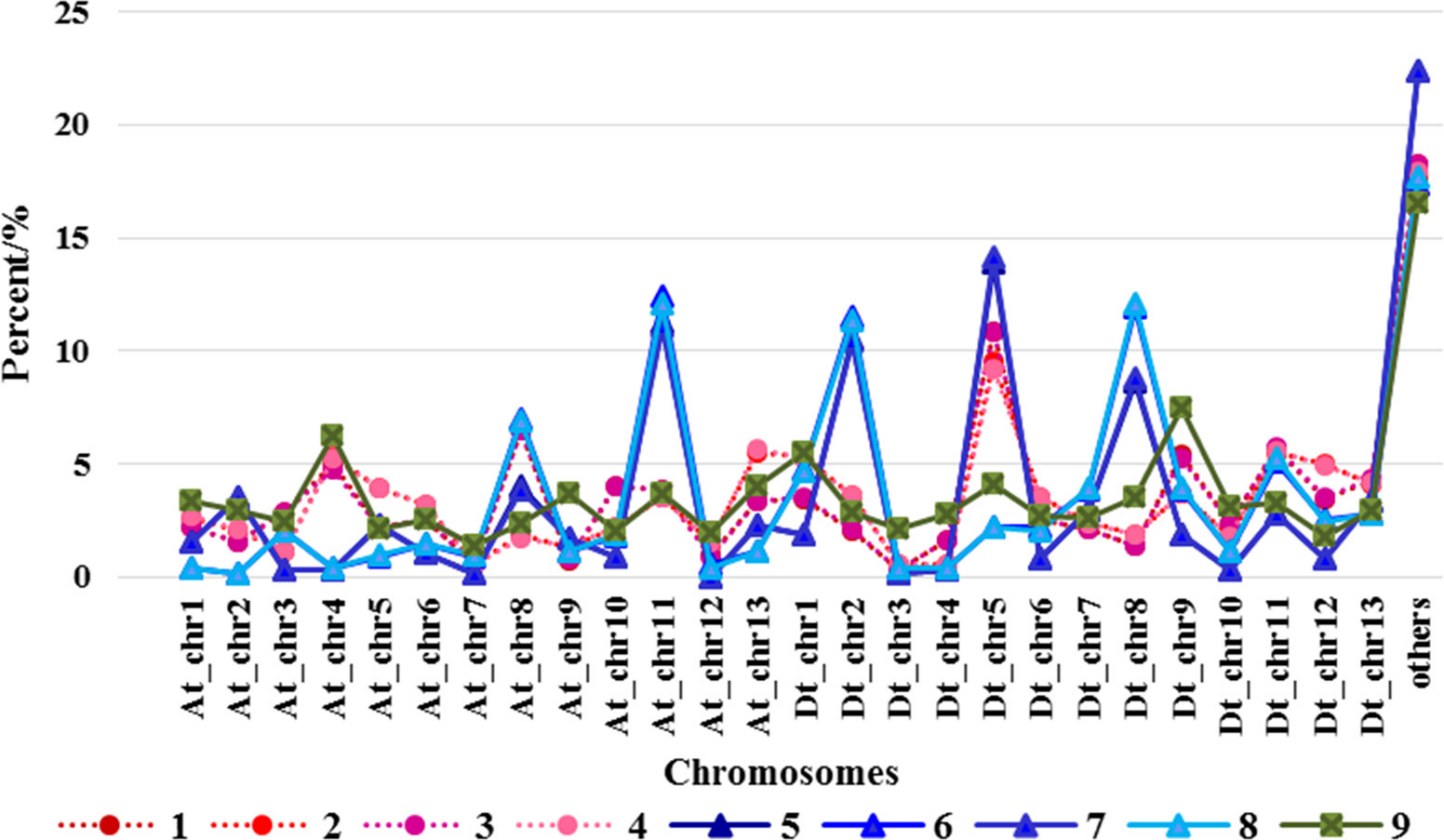
Distribution on the chromosomes of SNPv of eight varieties and chip SNP. 1~4: the salt sensitive varieties: Hengmian3, GK50, Xinyan96-48, ZhongS9612; 5~8: the salt tolerant varieties: CRI35, Zhong9807, CRI 44, Kanghuangwei164; 9: Illumina Cotton SNP 70K Chip.

Bowtie software was used to determine the location of SNPv in different varieties. SNPv numbers and proportion on different chromosomes were in statistics, and compared with those of SNP chips. As is shown in Fig 2, SNPv of eight varieties have significant differences with SNP chip in distribution on the chromosomes, suggesting that SNPv did not occur randomly. There is a similarity of SNPv distribution on chromosomes within salt-tolerant group and salt-sensitive group, and a difference of that between salt-tolerant group and salt-sensitive group. So the occurrence of SNPv may be associated with genetic background or salt tolerance of cotton. Fig 2 showed that the proportions of SNPv site on At_chr4,At_chr11,Dt_chr2,Dt_chr8 in salt-tolerant varieties are significantly different from those in salt-sensitive varieties.

#### Statistics of ST-SNPv and SS-SNPv

SNPv statistics of ST group and SS group showed that SNPv was mainly about A/G and C/T mutation, accounting for two-thirds of the four conversion types (Table 1).

#### GO analysis of genes containing chip-SNP, SS-SNPv and ST-SNPv

Bowtie software was used to align the probe sequence of chip SNP, SS-SNPv and ST-SNPv to the reference genome, to determine the chromosomal location of the SNP. The results showed that among 63,058 chip-SNP loci, 39,603 were aligned to the upland cotton genome, 1,025 and 310 out of SS-SNPv and ST-SNPv were aligned to the genome, respectively. BEDTools software (www.plob.org/2012/09/26/3748.html) was used for annotation and analysis of SNP, and results showed that 12,077 chip-SNP were aligned to 9,164 genes, 236 out of 1,025 SS-SNPv were on the genes, 79 out of 310 ST-SNPv were aligned to the genes.

**Fig 3:**
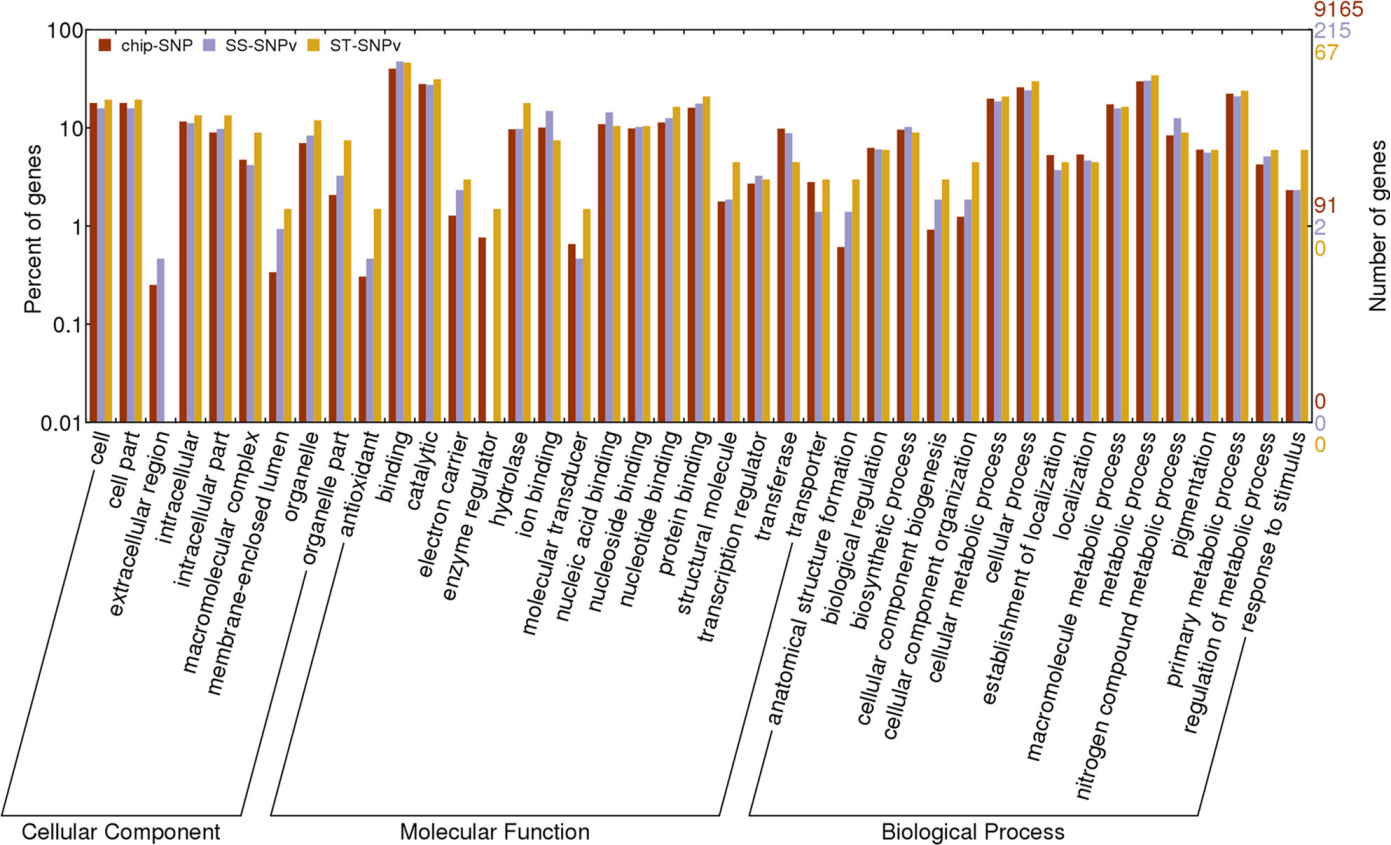
GO analysis of genes containing chip-SNP, SS-SNPv and ST-SNPv.

GO annotation of genes containing chip-SNP, SS-SNPv and ST-SNPv was performed, and 5,743, 140 and 43 genes were annotated on the GO terms, respectively. Statistical mapping using BGI WeGO (http://wego.genomics.org.cn/cgi-bin/wego/index.pl) found that in most GO terms, there is no significant difference between the percentages of these three groups of genes (Fig 3). But in binding (p = 0.029), ion binding (p = 0.021), nitrogen compound metabolic process (p = 0.002), there is a significant difference in the gene percentage of chip-SNP and SS-SNPv, and there is a significant difference in hydrolase activity (p = 0.024) of gene of chip-SNP and gene of ST-SNPv, indicating that SNPv was significantly enriched in genes in these terms. These above differences showed that in the test SNPv did not occur randomly, but with a certain bias, and this bias was different in salt-tolerant and salt-sensitive materials. SNPv in salt-tolerant materials tends to occur in the genes of hydrolase activity entries, while SNPv in salt-sensitive materials tends to occur in the genes of binding, ion binding and nitrogen compound metabolic process.

#### Comparison of methylation in SNPv rich region

**Fig 4:**
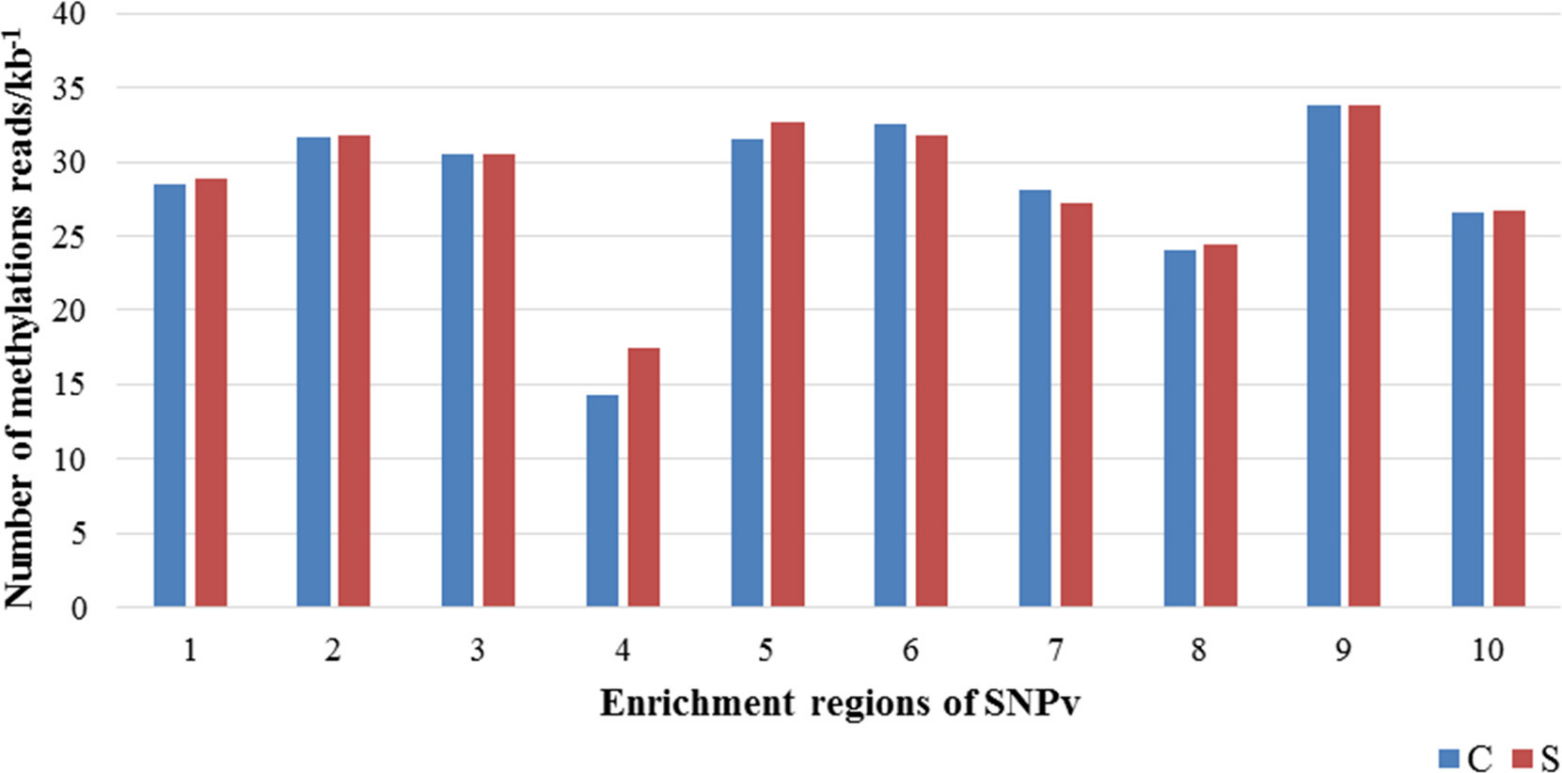
Numbers of methylation reads in SNPv rich region.

Combined with the DNA methylation immunoprecipitation data from the laboratory, the methylation status of SNPv enrichment region in the salt-tolerant material Zhong9807 was analyzed. According to the physical map obtained by Mapchart software, 10 relatively concentrated SNPv areas were screened (S2 Table), and they were located on 5 chromosomes. DNA methylation immunoprecipitation results showed that methylation distribution on the chromosome was quite different, and the number of methylation reads ranged from 0 to a few hundred per 10kb. Frequency of methylated reads in SNPv enrichment region was relatively stable, with methylation reads number being 30 or so per 10kb, which was relatively low, but close to the average level of the whole genome. Pre- and after- the salt stress, the frequency of methylation reads in SNPv rich region all changed (Fig 4), indicating that the occurrence of SNPv may be related to methylation. However, in the test, the frequency changes of methylation reads in SNPv rich region were not significant. Only SNP existing in the chip can be detected, so some SNPv consistent with the methylation enrichment may not be detected. Therefore the relationships between SNPv and methylation enrichment may require further experimental verification.

### Screening and analysis of SNPr

#### Screening of SNPr

The results showed that in eight materials, over 50,000 loci have been successfully genotyped. Remove the monomorphic loci (i.e. loci with the same genotypes in 8 materials, allowing for genotyping failure of two materials), and 7,087 polymorphic SNP loci were obtained, of which 4971 were annotated in the genome and covered all 26 chromosomes and 322 scaffolds. 1,804 SNP loci that may be related to salt tolerance were screened, of which 1,282 can be aligned to the upland cotton genome.

#### Distributions of SNPr on chromosomes

**Fig 5:**
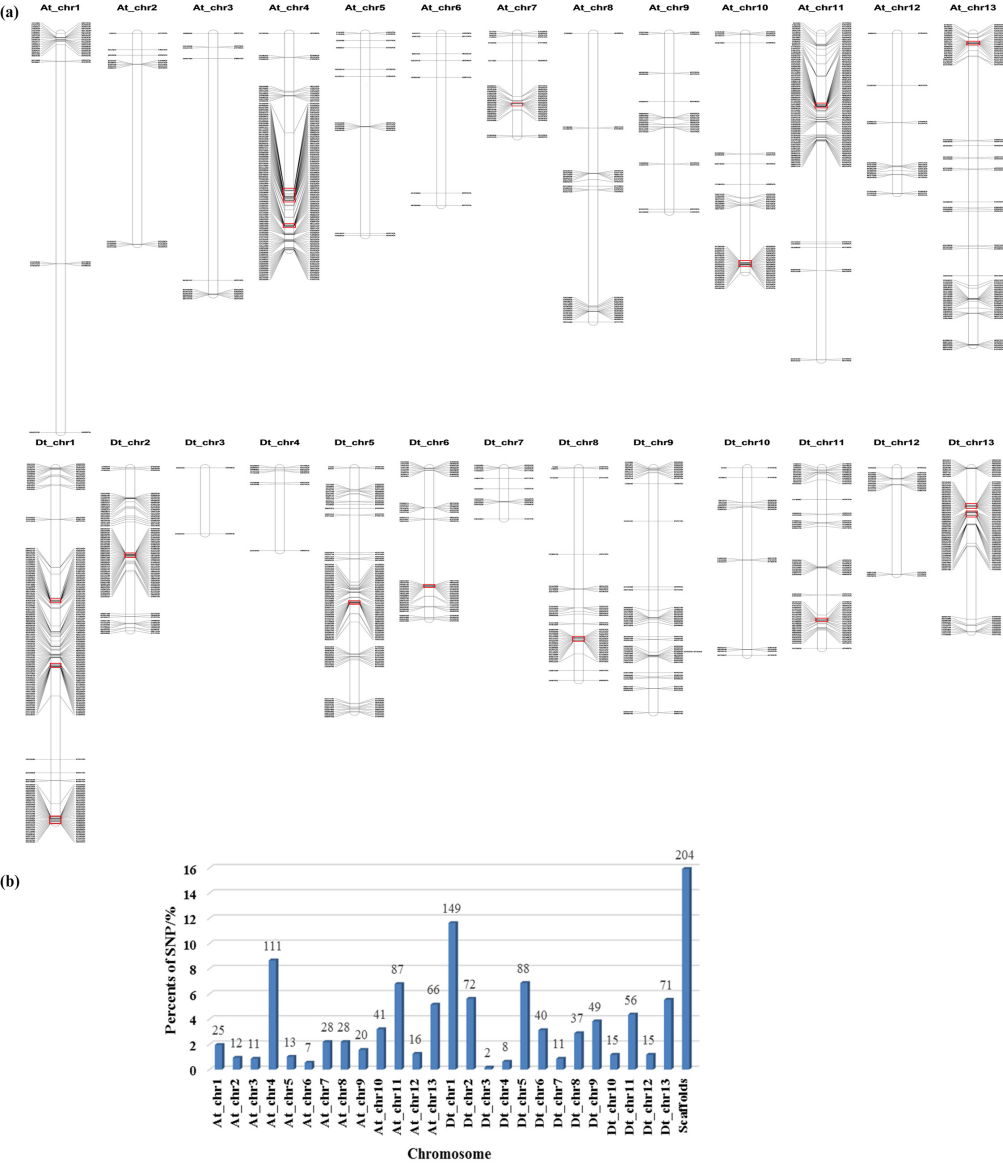
Locations (a) and distribution (b) of SNPr in different chromosomes. The boxes in Fig 5A are the rich region of SNPr.

Bowtie software was used to align probe of SNPr to the genome so as to determine its position on the chromosome, and their distribution on chromosomes was gathered. Mapchart mapping found that the distribution of SNPr on chromosome was uneven, and At_chr4 and Dt_chr1 had the largest distribution of SNPr (Fig 5).

#### GO analysis of genes in the rich region of SNPr

**Fig 6:**
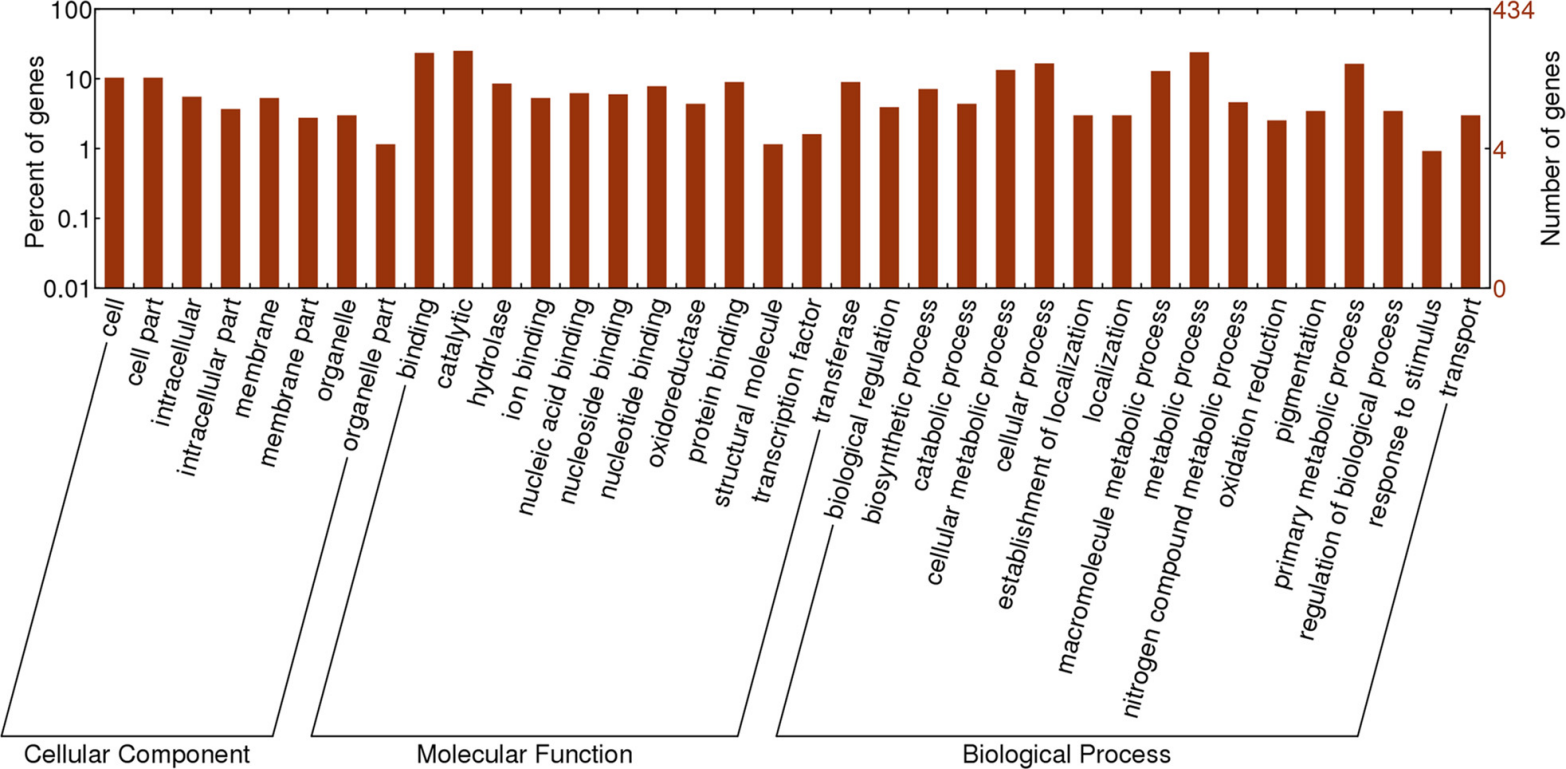
GO analysis of genes neighboring SNPr rich regions.

According to enrichment status of SNPr on chromosomes, 16 enriched zones of SNPr were found (Fig 5A and S3 Table), and 433 genes were found around the 16 enriched zones by BEDTools software, in which 198 genes were suitable for GO annotations. GO analysis found that they are mainly concentrated in GO terms including binding, metabolic process, primary metabolic processes, etc (Fig 6). Among them 75 genes in the membrane system, transcription factor activity, oxidoreductase, response to stimulus, transport, and ion binding were found in GO terms relevant to root’s response to salt tolerance [18], accounting for 43.46% of all annotated genes. Combined with the transcriptome of CRI35 subjected to salt, 278 differentially expressed genes in leaves were screened from 433 genes in SNPr enriched zones, accounting for 64.20%. In conclusion, there were 291 genes related to salt tolerance in 16 enriched zones of SNPr, acounting for 67.20%. This indicated that in the rich region of SNPr, salt tolerance-related genes were also relatively concentrated. These SNPr may regulate the salt tolerance of plants by regulating the expression of salt tolerance-related genes.

## Discussion

Under abiotic stress conditions, stoma closes, and CO_2_ uptake is inhibited and produces reactive oxygen species (ROS) such as H_2_O_2_, superoxide and single electron oxygen in chloroplasts, mitochondria and peroxisomes [19]. When the concentration of ROS exceeds the antioxidant defense capacity of organisms, it will produce oxidative stress, thereby damaging macromolecules such as proteins, lipids, carbohydrates and DNA [20]. It has been reported that both the purine and pyrimidine bases could be damaged by OH·, while ^1^O_2_ primarily attacks guanine [21]. DNA damage from ROS results in base deletion, pyrimidine dimers, cross-links, strand breaks and base modification [22]. What was more, polyunsaturated fatty acid (FUFA) peroxide will form some aldehydes, such as 4-hydroxy-2-nonenal (HNE) and malondialdehyde (MDA), hydroxyl and keto fatty acids. Lipid hydroperoxide will also decompose some active substances, such as lipid alkoxyl radicals, aldehydes, alkanes, lipid epoxides and alcohols [23, 24]. Most of these substances are high mutagen that will caused transversion or conversion of single bases through a series of physiological and biochemical processes [25]. These oxidation induced DNA damage was mainly repaired by base excision repair enzymes, and prior to cell replication, most damage has been repaired, while the damage that was not repaired becomes mutation [16]. So ROS may be one of the reasons why SNPv occurs under salinity stress.

Moreover, previous researches showed that the MDA accumulation at roots of salt-tolerant rice under salt stress was lower than that of salt-sensitive rice, indicating that ROS induced by salinity stress can cause higher rate of lipid peroxidation in salt-sensitive rice [26], which may be the reason why the numbers of SNPv in salt-sensitive cotton varieties were larger than those in salt-tolerant varieties in our study.

Studies had found that after salt stress, the methylation of salt-sensitive materials were higher than those of salt-tolerant materials [27], so did the SNPv in this study. 5mC at the methylated CpG site was prone to spontaneous deamination to form thymine, facilitating C/T or G/A mutation [28]. According to the statistics of the four kinds of SNPv types in this experiment, the variation types of G/A and C/T accounted for the highest proportion, which was in line with the conclusion above. Moreover, according to the results of the present experiment, we noted that there was a change in methylation reads number pre- and after- salt stress in SNPv-rich area, suggesting a potential link between methylation and SNPv. But more evidences were still needed to verify the association of SNPv and DNA methylation. DNMTs (DNA methyltransferases), which is an enzyme catalyzing CpG methylation after the completion of DNA repair [29], connecting DNA repair and methylation process, might be a focus to investigate the relationship between SNPv and methylation.

The current experiment adopted Illumina Cotton SNP 70K Chip to detect several upland cottons in different salt tolerance index so as to preliminarily screening SNPr and its adjacent genes. The results showed that most genes in rich region of SNPr were differentially expressed, which suggested that SNPr was likely to regulate the salt tolerance by controlling the expression of salt tolerance-associated genes. In fact, many of the SNP relevant to traits plays a role by regulating the expression of target genes, and most of them were in the gene or upstream 0 to 2000bp [10, 30–32]. Therefore, SNP within 2000bp on the differentially expressed genes or the upstream region is more likely to be relevant to salt tolerance and will be the focus of future research.

Advantageous alleles spread and deleterious or poorly adapted alleles were eliminated by natural selection [33]. The alleles that is favored by natural selection can originate from two sources: new mutations and pre-existing alleles [34]. Advantageous alleles finally were fixed and conferred the adaptation of organisms to different environments [35]. In our study, a lot of SNPv were found pre- and after- salt stress in cotton genome, which may be one source of mutations for natural selection. And the SNPr may be the result of fixation of advantageous mutations.

**Fig 7:**
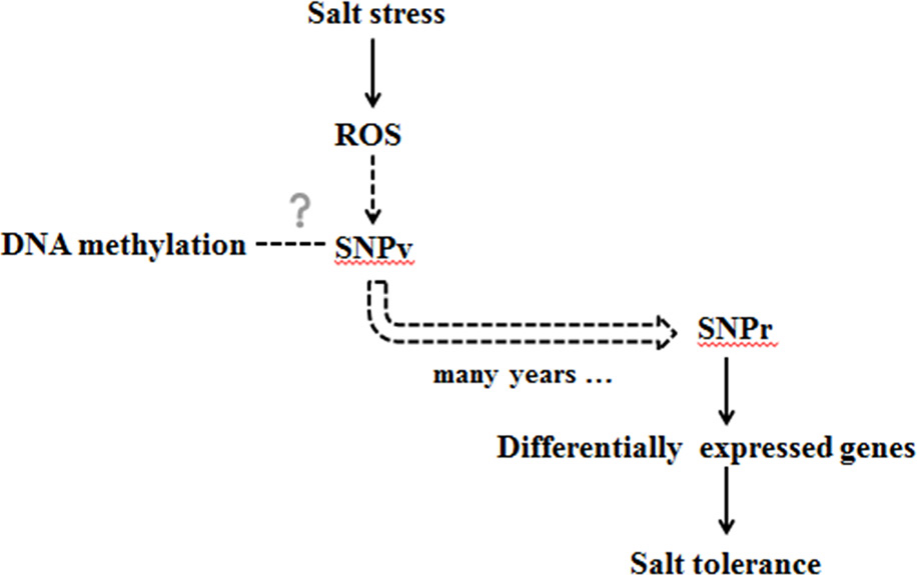
The possible relation of ROS, DNA methylation, SNPv and SNPr.

According all above, the connection of ROS, SNPv, DNA methylation, SNPr and genes could be elaborated as below (Fig 7): (1) ROS may be one of the sources of SNPv; (2) there is a potential link between SNPv and DNA methylation; (3) SNPr may be the result of natural selection from SNPv; (4) SNPr plays a role in salt tolerance by regulating the expression of target genes.

## Conclusions

Our data revealed that SNPv may be induced by salinity stress, and the induction efficiency of salt stress on SNPv was different among cotton varieties with different salt tolerance index. The induction of salt stress on SNPv was obviously biased. SNPv induced by salt stress may be related to the methylation changes under salinity stress. 1,282 SNPr were screened in our study and they may influence salt tolerance of plants by affecting the expression of salt tolerance-related genes.

## Supporting Information

S1 Table The experimental varieties and their salt tolerance index. (DOCX)

S2 Table SNPv rich regions and their numbers of methylation reads. (DOCX)

S3 Table Numbers and frequency of SNPr in SNPr rich regions. (DOCX)
