# Supplementary material for: Mining and Analysis of SNP in Response to Salinity Stress in Upland Cotton (Gossypium hirsutum L.)
Source: PLoS One. 2016 Jun 29;11(6):e0158142. doi: 10.1371/journal.pone.0158142 (PMC4927152; doi:10.1371/journal.pone.0158142)
Supplement: S1 Table — (DOCX) [file pone.0158142.s001.docx]

| Group | Varieties | Salt tolerance index/% |
| --- | --- | --- |
| Salt-tolerant group | CRI35 | 65.04 |
|  | Kanghuangwei164 | 56.19 |
|  | Zhong9807 | 55.20 |
|  | CRI44 | 50.50 |
| Salt-sensitive group | Hengmian3 | 48.21 |
|  | GK50 | 40.20 |
|  | Xinyan96-48 | 34.90 |
|  | ZhongS9612 | 24.80 |

Table S1 The experimental varieties and their salt tolerance index
