# Supplementary material for: Mining and Analysis of SNP in Response to Salinity Stress in Upland Cotton (Gossypium hirsutum L.)
Source: PLoS One. 2016 Jun 29;11(6):e0158142. doi: 10.1371/journal.pone.0158142 (PMC4927152; doi:10.1371/journal.pone.0158142)
Supplement: S2 Table — (DOCX) [file pone.0158142.s002.docx]

| Region | Chromosome | Start and end  (5’-3’) | Length/kb | Numbers of methylation reads  in control(C)/kb^-1^ | Numbers of methylation reads  in salt-treated material(S)/kb^-1^ |
| --- | --- | --- | --- | --- | --- |
| 1 | At_chr11 | 19253800-19866304 | 612.504 | 28.46 | 28.81 |
| 2 | At_chr11 | 22473891-23997297 | 1523.406 | 31.69 | 31.78 |
| 3 | At_chr11 | 27361833-28827123 | 1465.29 | 30.58 | 30.50 |
| 4 | Dt_chr2 | 28550722-29441587 | 890.865 | 14.33 | 17.48 |
| 5 | Dt_chr2 | 42392793-42676629 | 283.836 | 31.48 | 32.71 |
| 6 | Dt_chr2 | 45151948-45644244 | 492.296 | 32.56 | 31.80 |
| 7 | Dt_chr8 | 812601-865441 | 52.840 | 28.12 | 27.23 |
| 8 | Dt_chr8 | 44571486-45824443 | 1252.957 | 24.07 | 24.42 |
| 9 | Dt_chr12 | 18819658-18977159 | 157.501 | 33.85 | 33.82 |
| 10 | Dt_chr13 | 8241871-8475260 | 233.389 | 26.56 | 26.68 |

Table S2 SNPv rich regions and their numbers of methylation reads
