# Supplementary material for: Mining and Analysis of SNP in Response to Salinity Stress in Upland Cotton (Gossypium hirsutum L.)
Source: PLoS One. 2016 Jun 29;11(6):e0158142. doi: 10.1371/journal.pone.0158142 (PMC4927152; doi:10.1371/journal.pone.0158142)
Supplement: S3 Table — (DOCX) [file pone.0158142.s003.docx]

| Chromosome | Start-End (5’-3’) | Numbers of SNPr | Length of enrichment region/kb | frequency of SNPr/kb^-1^ |
| --- | --- | --- | --- | --- |
| At_chr4 | 62409266-65317101 | 35 | 2907.84 | 0.0120 |
| At_chr4 | 71566264-72004374 | 18 | 438.11 | 0.0411 |
| At_chr7 | 18569698-18989664 | 11 | 419.97 | 0.0262 |
| At_chr10 | 91786208-92612343 | 23 | 826.14 | 0.0278 |
| At_chr11 | 19253800-19866304 | 21 | 612.50 | 0.0343 |
| At_chr13 | 9208400-9436054 | 13 | 227.65 | 0.0571 |
| Dt_chr1 | 37268243-37982658 | 17 | 714.42 | 0.0238 |
| Dt_chr1 | 54144457-55113634 | 19 | 969.18 | 0.0196 |
| Dt_chr1 | 94563303-96236642 | 32 | 1673.34 | 0.0191 |
| Dt_chr2 | 28550722-29441587 | 31 | 890.87 | 0.0348 |
| Dt_chr5 | 35483773-35909135 | 16 | 425.36 | 0.0376 |
| Dt_chr6 | 34053382-34451503 | 12 | 398.12 | 0.0301 |
| Dt_chr8 | 45000797-45774943 | 15 | 774.15 | 0.0194 |
| Dt_chr11 | 47613151-47799635 | 11 | 186.48 | 0.0590 |
| Dt_chr13 | 18223201-18658005 | 18 | 434.80 | 0.0414 |
| Dt_chr13 | 19856383-20942212 | 14 | 1085.83 | 0.0129 |

Table S3 Numbers and frequency of SNPr in SNPr rich regions
